# Ssc-miR-92b-3p Regulates Porcine Trophoblast Cell Proliferation and Migration via the *PFKM* Gene

**DOI:** 10.3390/ijms232416138

**Published:** 2022-12-17

**Authors:** Yongzhong Wang, Chen Zhou, Fanming Meng, Qun Hu, Yue Ding, Xiaoliang Wang, Ting Gu, Zicong Li, Zhenfang Wu, Linjun Hong, Gengyuan Cai

**Affiliations:** 1 National Engineering Research Center for Breeding Swine Industry, College of Animal Science, South China Agricultural University, Guangzhou 510642, China; 2 Guangdong Provincial Key Laboratory of Agro-Animal Genomics and Molecular Breeding, South China Agricultural University, Guangzhou 510642, China; 3 State Key Laboratory of Livestock and Poultry Breeding, Guangdong Key Laboratory of Animal Breeding and Nutrition, Institute of Animal Science, Guangdong Academy of Agricultural Sciences, Guangzhou 510640, China; 4 Yunfu Subcenter of Guangdong Laboratory for Lingnan Modern Agriculture, Yunfu 527300, China

**Keywords:** ssc-miR-92b-3p, *PFKM*, embryo implantation

## Abstract

Embryo implantation, the pivotal stage of gestation, is fundamentally dependent on synchronous embryonic development and uterine receptivity. In the early gestation period, the uterus and conceptus secrete growth factors, cytokines, and hormones to promote implantation. Circulating exosomal miRNAs are potential indicators of normal or complicated gestation. Our previous study revealed that pregnant sows’ serum exosomes had upregulated miR-92b-3p expression compared to non-pregnant sows, and that the expression level progressively increased during early gestation. The present study’s findings indicate that, compared to the ninth day of the estrous cycle (C9), pregnant sows had upregulated miR-92b-3p expression in the endometrium and embryos during the implantation stage ranging from day 9 to day 15 of gestation. Additionally, our results demonstrate that miR-92b-3p promotes the proliferation and migration of Porcine Trophoblast Cells (PTr2). Dual-Luciferase Reporter (DLR) gene assay, real-time fluorescent quantitative PCR (RT-qPCR), and Western blotting (WB) confirmed the bioinformatics prediction that phosphofructokinase-M (*PFKM*) serves as a target gene of miR-92b-3p. Notably, interference of *PFKM* gene expression markedly promoted PTr2 proliferation and migration. Furthermore, mice with downregulated uterine miR-92b-3p expression had smaller rates of successful embryo implantation. In summary, miR-92b-3p putatively modulates embryo implantation by promoting PTr2 proliferation and migration via its target gene *PFKM*.

## 1. Introduction

Spontaneous embryonic loss is a long-standing major challenge impacting commercial swine production worldwide, with 20–45% of total embryonic losses occurring during gestation [1] and approximately 20–30% during the early stage of implantation (between days 12 and 30 of gestation) [2]. Around the 12th day of gestation, porcine embryos migrate from one uterine horn to the other to accommodate each other and successfully attach to the receptive uterine epithelium [3]. Steroid hormones [4] prepare the endometrium for embryo implantation, while growth factors, prostaglandins, and exosomes [5] facilitate specific dialogs.

During gestation, the placental tissue permits the maternal–fetal exchange of nutrients, oxygen, and waste products, provides a natural protective barrier for fetal growth and development, and produces essential hormones and growth factors to support gestational health [6]. Exosomes are nano-sized (30–150 nm in diameter), membrane-bound extracellular vesicles secreted via exocytosis by diverse cell types [7,8,9]. In mammals, exosomes or microvesicles are present in most body fluids, such as saliva [10], urine [11], semen [12], porcine uterine cavity fluid [13], and plasma [14]. These molecules comprise metabolites, mRNAs, lipids, functional proteins, and miRNAs with consequential roles in microenvironment modulation and intercellular communication [15]. MicroRNAs (miRNAs), short RNA molecules spanning from 18 to 25 nucleotides in size, are crucial modulators of gene expression and regulate gene expression post-transcriptionally. They regulate the growth, differentiation, development, and apoptosis of various cells [16]. Our previous study indicated upregulated miR-92b-3p in serum exosomes isolated from pregnant sows in the early stage of pregnancy [17]. Stacy M Yadava et al. [18] discovered that, in pregnant women, umbilical artery-derived exosomes were internalized by the placenta; furthermore, compared with early cesarean delivery, delivery at term was associated with significantly increased placental miR-92b-3p expression. Zhou [17] and Hua [19] et al. reported that miR-92b-3p expression was upregulated during the embryo implantation stage, however, the role of miR-92b-3p during the embryo implantation process remains elusive.

*PFKM* is a critical rate-limiting enzyme involved in the Embden–Meyerhof pathway [20]. Early embryonic development occurs in a hypoxic environment that enables anaerobic glycolysis, and placental cells are considered as potential promoters of glycolysis and inhibitors of mitochondrial metabolism [21,22]. However, as placental blood vessels gradually mature, the placenta, which had initially adapted to hypoxia, starts developing in an oxygen-rich environment. Excessive glycolysis promotes reactive oxygen species (ROS) production in the placenta, increases oxidative stress exposure, and eventually impedes maternal homeostasis and fetal growth and development [23,24]. Glycolysis inhibition redirects metabolism toward the oxidative pentose phosphate pathway, which supplies cells with pentose for the biosynthesis of nucleotides and nucleic acids and generates nicotinamide adenine dinucleotide phosphate (NADPH) against oxidative stress [25]. Hence, effective regulation of placental metabolism is essential for gestational health [26].

Furthermore, previous studies have confirmed the crucial role of miRNAs in the identification of pregnancy and maternal–fetal communication and, by extension, as biomarkers of early pregnancy in mammals [27]. However, how maternal circulating exosomal miRNA regulates embryo implantation remains obscure. In this study, embryonic tissues’ miR-92b-3p expression gradually increased on days 9, 12, and 15 of pregnancy. Further investigation revealed that ssc-miR-92b-3p enhanced PTr2 proliferation and migration via the *PFKM* gene and was beneficial for embryo implantation. Our results provide valuable insights into the mechanism by which ssc-miR-92b-3p regulates embryo implantation.

## 2. Results

### 2.1. The Expression Profile of ssc-miR-92b-3p

**Figure 1:**
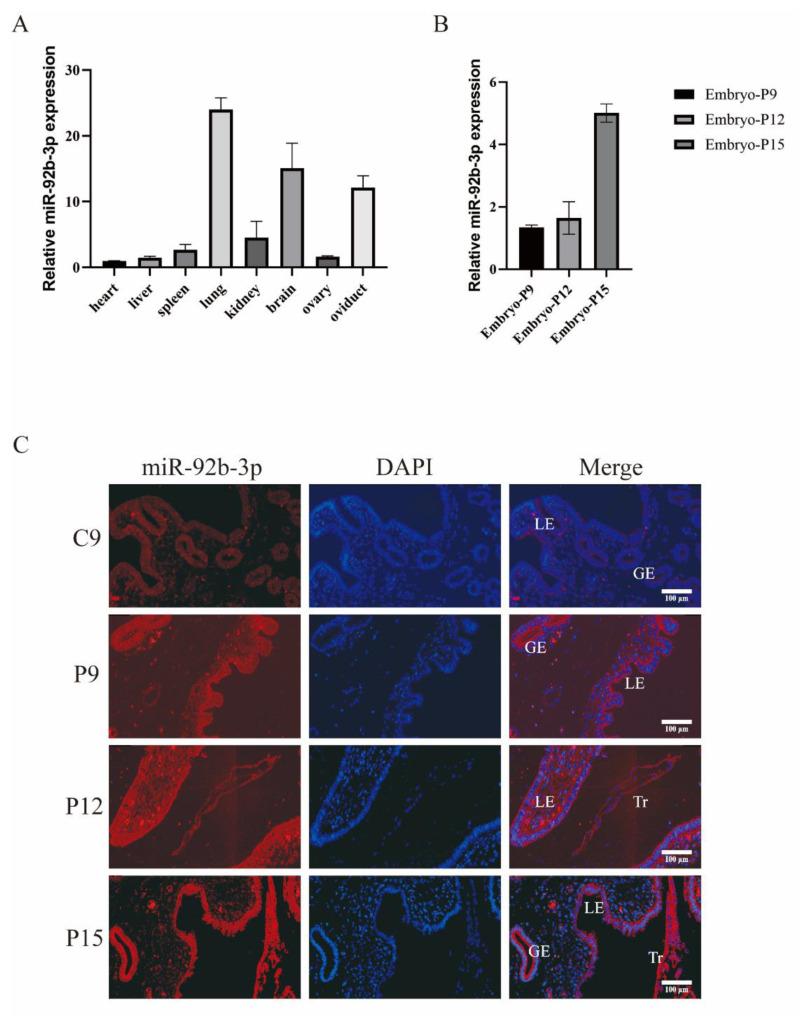
The expression profile of ssc-miR-92b-3p. (**A**) Real-time fluorescence quantification of miR-92b-3p expression in different pig tissues. (**B**) miR-92b-3p expression gradually increased in embryonic tissue on days 9, 12, and 15 of pregnancy (P9, 12, and 15). (**C**) miR-92b-3p (red fluorescence) expression gradually increased in endometrial tissue on day 9 of the estrus cycle (C9), and in embryonic tissue on days 9, 12, and 15 of pregnancy (P9, 12, and 15). GE: glandular epithelial tissue, LE: luminal epithelial tissue, and Tr: Trophoblast Cell. Scale bar 100μm.

Ssc-miR-92b-3p expression in several pig tissues was detected by real-time quantitative PCR (RT-qPCR); the results demonstrated widespread expression in a range of pig tissues (Figure 1A). To determine whether miR-92b-3p was associated with embryo attachment, qPCR of embryo tissues from early-pregnancy sows was performed; miR-92b-3p in embryonic tissues was progressively upregulated during days 9, 12, and 15 of pregnancy (P9, 12, and 15) (Figure 1B). In situ hybridization results also indicated that compared with the expression level on the ninth day of the estrous cycle (C9), the endometrial tissue miR-92b-3p was upregulated on days 9, 12, and 15 of pregnancy (P9, 12, and 15). (Figure 1C) (Table S1 and Figure S1).

### 2.2. The Role of miR-92b-3p in Porcine Trophoblast Cells

**Figure 2:**
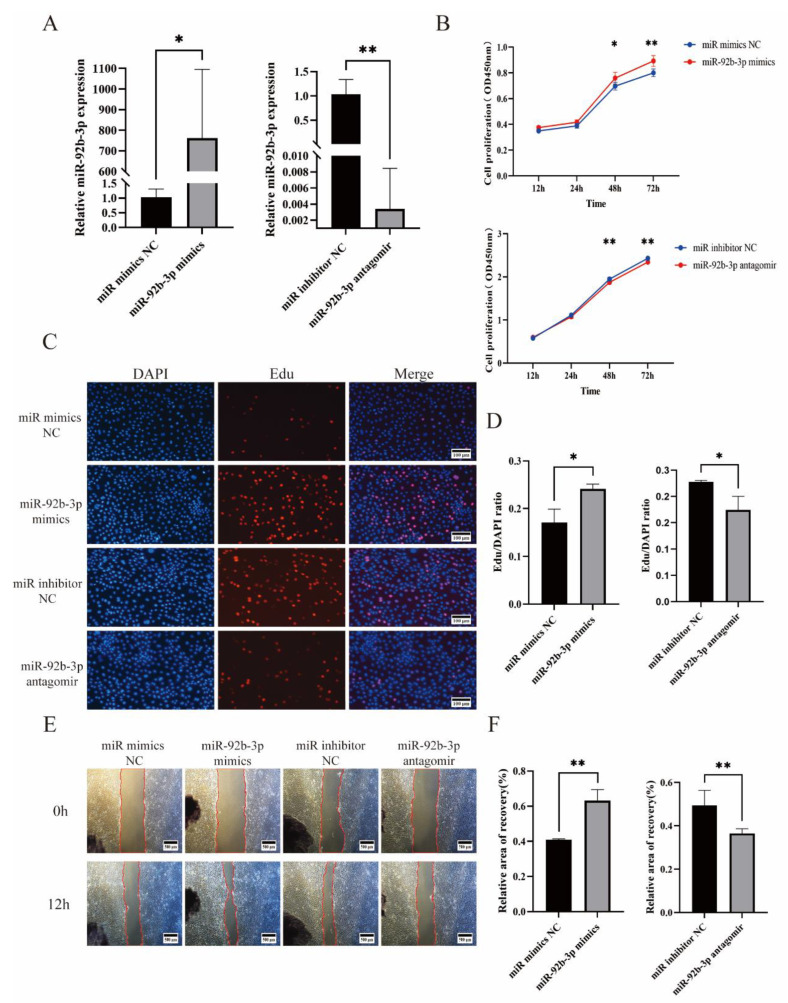
Effect of miR-92b-3 on PTr2 proliferation and migration. (**A**) Transfection of miR-92b-3p mimics into PTr2 induced a significant upregulation of miR-92b-3p expression; transfection of miR-92b-3p inhibitors induced an opposite effect. (**B**–**D**) CCK-8 and Edu results demonstrated that miR-92b-3p overexpression promoted PTr2 proliferation, and inhibition of miR-92b-3p expression inhibited PTr2 proliferation. (**E**, **F**) Elevated miR-92b-3p promoted PTr2 migration; reduced miR-92b-3p inhibited PTr2 migration. Data are expressed as the means ± SEM of three experiments. (* *p* < 0.05; ** *p* < 0.01).

To explore the role of miR-92b-3p in porcine trophoblast cells (PTr2), either ssc-miR-92b-3p mimics or antagomir were transfected into PTr2. RT-qPCR results revealed that miR-92b-3p expression was significantly upregulated after transfection with ssc-miR-92b-3p mimics; conversely, transfection with the antagomir downregulated miR-92b-3p expression (Figure 2A). Cell Counting Kit-8 (CCK-8) and the Edu assay were utilized to evaluate PTr2 cell proliferation. Cell Counting Kit-8 (CCK-8) and Edu assay results indicated a positive correlation between ssc-miR-92b-3p and PTr2 proliferation, whereby alterations in ssc-miR-92b-3p expression had a proportional and significant impact on PTr2 proliferation (Figure 2B–D). Furthermore, wound healing assay results demonstrated that ssc-miR-92b-3p elevation significantly promoted PTr2 migration, while ssc-miR-92b-3p downregulation significantly inhibited PTr2 migration (Figure 2E,F). In conclusion, our findings indicate that miR-92b-3p promotes PTr2 proliferation and migration.

### 2.3. Ssc-miR-92b-3p Directly Targets the 3’-UTR of PFKM in PTr2

**Figure 3:**
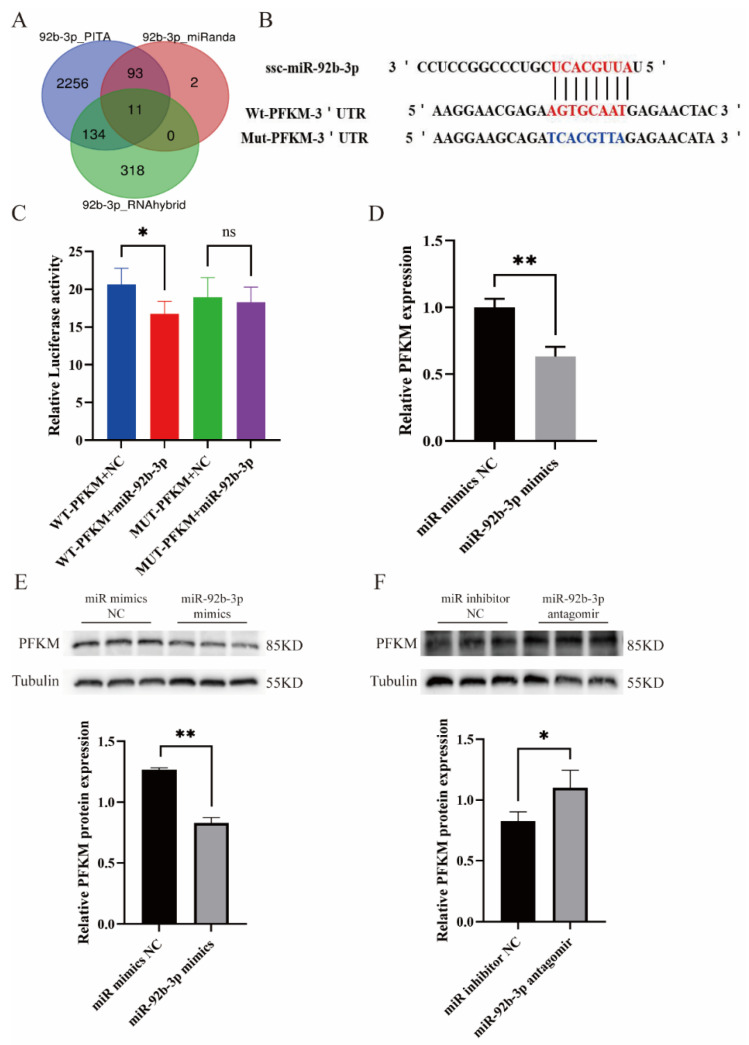
miR-92b-3p targets and regulates the *PFKM* gene in PTr2. (**A**) The target genes of miR-92b-3p as predicted by 3 databases. (**B**) Prediction of binding sites and mutation sites of miR-92b-3p to the 3′UTR of porcine *PFKM* gene. (**C**) The relative luciferase activity was significantly reduced after co-transfection with wild-type (WT) *PFKM*3′UTR and miR-92b-3p mimics, while no significant change in relative luciferase activity was observed after co-transfection with mutant (MUT) *PFKM*3′UTR. (**D**) qPCR analysis indicated that *PFKM* mRNA expression in PTr2 decreased after transfection with miR-92b-3p mimics. (**E**,**F**) Protein blot analysis revealed that *PFKM* protein expression in PTr2 decreased after transfection with miR-92b-3p mimics; the reverse was observed following transfection with miR-92b-3p antagomir. Data are expressed as the means ± SEM of three experiments. (ns: no significant difference; * *p* < 0.05; ** *p* < 0.01).

Target gene prediction for ssc-miR-92b-3p was conducted using the target gene prediction software RNAhybrid, PITA, and miRanda, respectively (Figure 3A). Subsequent to screening, *PFKM* was selected for further investigation. Wild-type (WT) and Mutant (MUT) luciferase reporter plasmids carrying *PFKM* 3’-UTR were constructed (Figure 3B). The relationship between PKFM and miR-92b-3p was further verified using a dual-luciferase reporter assay, which revealed significantly reduced luciferase activity when miR-92b-3p mimics were co-transfected with a wild-type luciferase reporter plasmid (pmirGLO-*PFKM*-WT); after co-transfection with the mutant luciferase reporter plasmid (pmirGLO-*PFKM*-MUT), luciferase activity showed no significant change (Figure 3C). These findings demonstrate that miR-92b-3p directly targets *PFKM*. Moreover, the real-time fluorescence quantitative PCR (qPCR) and Western blot (WB) assays indicated downregulated *PFKM* RNA and protein expression levels post-transfection with miR-92b-3p mimics in PTr2 (Figure 3D,E); there was no significant difference in RNA level following miR-92b-3p antagomir transfection, but protein expression was significantly upregulated (Figure 3F).

### 2.4. The Role of PFKM in PTr2

**Figure 4:**
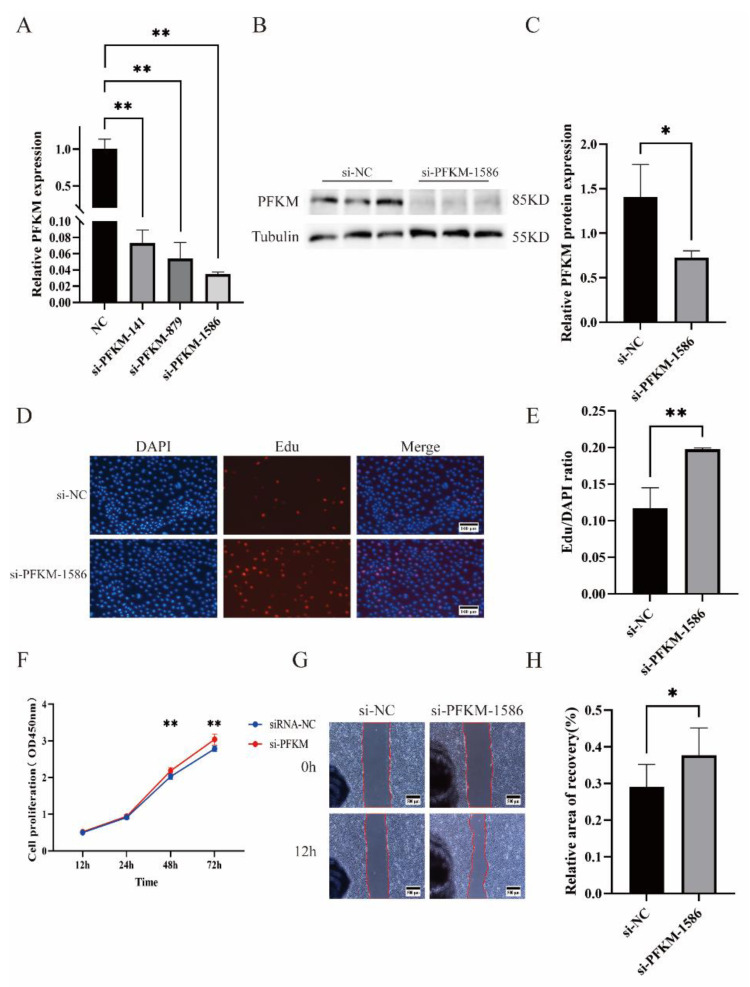
Functional effects of interference on *PFKM* gene expression in PTr2. (**A**) Three interfering fragments of the *PFKM* gene were constructed, of which si-*PFKM*-1586 demonstrated the most potent interference and was therefore used in all subsequent experiments. (**B**,**C**) Protein blot analysis revealed that si-*PFKM*-1586 significantly interferes with *PFKM* protein expression. (**D**–**F**) CCK-8 and Edu results showed that interfering with the *PFKM* gene promotes PTr2 proliferation. (**G**,**H**) Interfering with *PFKM* gene expression promotes PTr2 migration. Data are expressed as the means ± SEM of three experiments. (* *p* < 0.05; ** *p* < 0.01).

To investigate the functional effects of *PFKM* on PTr2, we designed three interfering fragments at different positions for the *PFKM* gene and transfected these three si-*PFKM* into PTr2. All three interfering fragments downregulated *PFKM* gene expression, but si-*PFKM*-1586 interfered with *PFKM* gene expression most significantly (Figure 4A); significant interference was equally observed for protein levels (Figure 4B,C). Therefore, si-*PFKM*-1586 was selected for subsequent functional validation investigations. CCK-8 (Figure 4F) and Edu (Figure 4D,E) experiments demonstrated that si-*PFKM* significantly promoted PTr2 proliferation, and cell-scratch assays indicated that interference of *PFKM* expression significantly promoted PTr2 migration (Figure 4G,H).

### 2.5. Si-PFKM Rescued the Influence of miR-92b-3p Antagomir in PTr2

**Figure 5:**
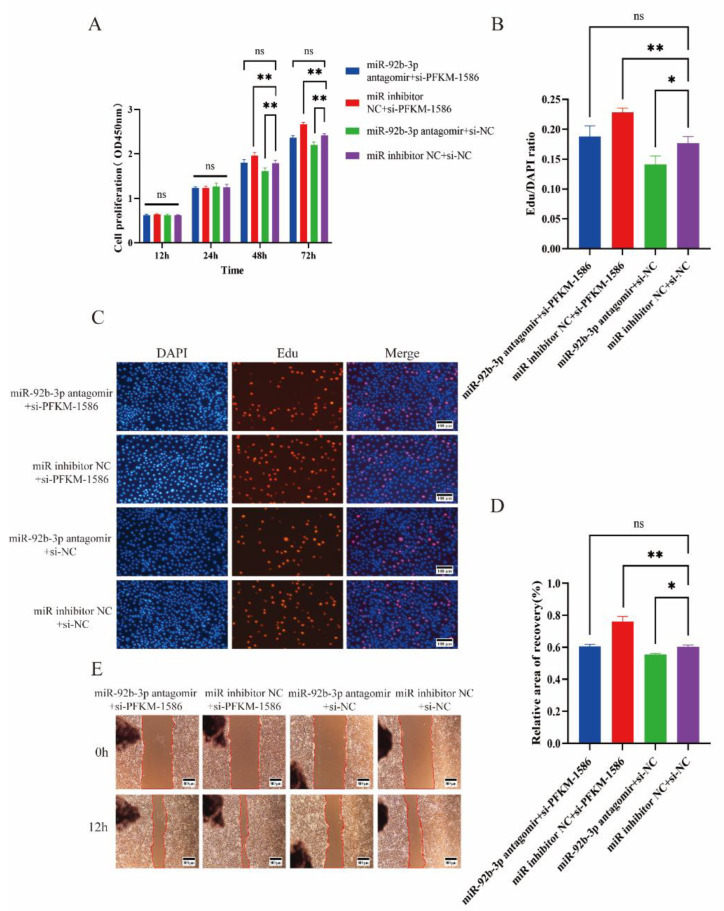
Knockdown of *PFKM* can rescue the effect of miR-92b-3p antagomir on the proliferation and migration of PTr2. (**A**–**C**) si-*PFKM*-1586 reversed the proliferation of PTr2 inhibited by miR-92b-3p antagomir. (**D**,**E**) The reduced number of cell migrations caused by antago-miR-92b-3p was partially rescued by si-*PFKM*-1586. Data are the means ± SEM of three experiments. (ns: no significant difference; * *p* < 0.05; ** *p* < 0.01).

Previous experiments revealed that miR-92b-3p targets and regulates the *PFKM* gene. To verify whether ssc-miR-92b-3p affects PTr2 proliferation and migration by targeting *PFKM*, si-*PFKM* was transfected into PTr2 to rescue the effect of miR-92b-3p antagomir on PTr2. The co-transfected miR-inhibitor NC and si-NC were used as the control group, and the results indicated that the groups co-transfected with miR-92b-3p antagomir and si-NC had inhibited cell proliferation (Figure 5A–C) and migration (Figure 5D,E) compared with the control group. Co-transfection with miR-inhibitor NC and si-*PFKM*-1586 promoted cell proliferation and migration, while for co-transfection with miR-92b-3p antagomir and si-*PFKM*-1586, the difference was not significant compared with the control group.

### 2.6. Inhibition of miR-92b-3p Hindered Mouse Embryo Implantation in a Mouse Model

**Figure 6:**
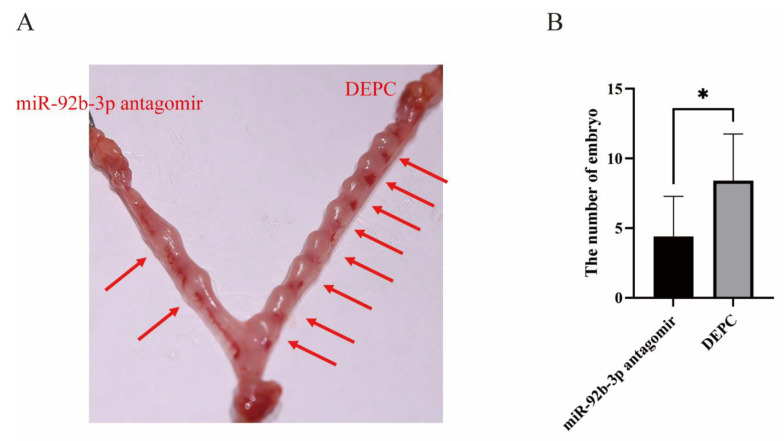
The function of miR-92b-3p in the mouse embryo attachment process. (**A**,**B**) Comparison of the number of implantation sites in mice injected with miR-92b-3p antagomir in one uterine horn (left side) and DEPC water in the other uterine horn (right side). The statistical significance of differences between means was assessed using paired *t*-tests (* *p* < 0.05).

We further validated the role of miR-92b-3p in embryo attachment by utilizing an in vivo mouse model. On day 3 of gestation, miR-92b-3p antagomir was injected into one horn of the mouse uterus, and DEPC water was injected into the other horn of the uterus as a control. On day 7 of gestation, the mice were dissected and observed for embryo attachment; the number of embryos attached on the miR-92b-3p antagomir injection side of the uterus was significantly smaller than that in the control group on the other side (Figure 6A,B).

## 3. Discussion

Embryo implantation is essential to the gestational process and strongly correlates with litter weight and size [28,29]. Successful embryo implantation partially depends on the information exchanged between embryos and the pregnant sow [30]. Compelling evidence indicates that miRNAs are involved in the regulation of embryonic implantation and development [18,31,32,33]. In our previous study, miR-92b-3p expression was upregulated in the serum of pregnant sows, and the expression level was continuously upregulated during the process of embryo implantation [17]; however, the mechanism underlying miR-92b-3p modulation during early gestation remained elusive. Therefore, this study attempts to elucidate the mechanism of miR-92b-3p involvement in embryo implantation.

MiRNAs are confirmed to be vital for embryo implantation. Zhou et al. found that exosome concentration increased in the plasma of pregnant sows and that, in the fatter ones, plasma exosomes contained a high level of miR-221 that inhibited endothelial cell migration and angiogenesis by acting on the ANGPTL2 gene [31]. Upregulated miR-92b-3p expression reportedly supports cancer cell proliferation and migration in various human cancers [34,35]. On the other hand, miR-92b-3p is significantly enriched in human embryonic stem cells and is strongly associated with the regulation of embryonic stem cell proliferation [36]. In the present study, miR-92b-3p expression was significantly upregulated in the porcine endometrium and embryonic tissue and was significantly elevated in the endometrial tissue of pregnant sows compared with that of non-pregnant sows, with a progressive increase throughout the gestation period. Hence, miR-92b-3p is strongly correlated with embryo implantation. The results demonstrated that miR-92b-3p promotes PTr2 proliferation and migration. On the 12th day of gestation, porcine embryos undergo a swift transition from spherical to filamentous forms, and the specific sites of implantation are determined. During this process of morphological and functional alterations, embryo death reaches up to 30–40% due to epiboly failure and insufficient contact between the embryonic trophectoderm and the epithelial cells on the endothelium [37]. This implies that miR-92b-3p expression is upregulated during gestation to promote PTr2 proliferation and migration, thereby facilitating embryo implantation. After injection of a miR-92b-3p inhibitor into one side of the uterine horns of mice, the antago-miR-92b-3p group had significantly fewer implanted embryos than the control group.

In the preimplantation period, miRNAs modulate the implantation and growth of embryos by regulating target genes [31,32,38]. *PFKM* is a kinase that catalyzes the phosphorylation of D-fructose 6-phosphate to fructose 1,6-bisphosphate, as well as the rate-limiting step of glycolysis [39]. During embryo implantation, the uterine cavity contains higher concentrations of glucose and fructose, two major energy substrates that fuel fetal growth and development [40]. In this study, we utilized three online tools, miRanda, RNAhybrid, and Targetscan, to analyze the suitability of *PFKM* as a potential target gene for ssc-miR-92b-3p, while the DLR gene assay, RT-qPCR, and WB analysis demonstrated the inhibitory effects of miR-92b-3p on *PFKM* mRNA and protein expression. Studies have reported that, under hypoxic conditions, *PFKM* Ser529 achieves glycosylation through post-translation modification of acetylglucosamine to inhibit *PFKM* activity and reroutes glucose flux away from the pentose phosphate pathway; this results in an increased D-ribose 5-phosphate level supportive of rapid cancer cell growth through DNA and protein synthesis [41]. However, the role of *PFKM* in PTr2 is still unclear. In this study, subsequent to PTr2 treatment with small molecule inhibitors of *PFKM* (si-*PFKM*), it was observed that suppressing *PFKM* significantly promoted the proliferation and migration of PTr2. Successful embryo implantation and placental development require the ability of embryonic trophoblast cells to proliferate and migrate [42]. Furthermore, we demonstrated that interfering with *PFKM* expression reversed the effect of miR-92b-3p antagomir on PTr2 by conducting co-transfection experiments. These findings suggest that ssc-miR-92b-3p regulates the proliferation and migration of PTr2 by targeting *PFKM*.

**Figure 7:**
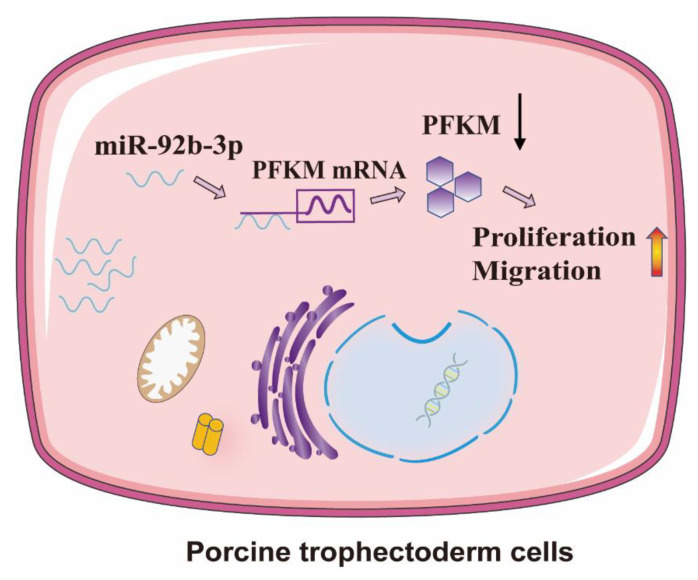
ssc-miR-92b-3p regulates porcine trophoblast cell proliferation and migration via the *PFKM* gene.

In conclusion, miR-92b-3p expression is progressively upregulated in the endometrium and embryos from day 9 to day 15 of gestation, and the endometrial expression level is consistently higher in pregnant sows than on the ninth day of the estrous cycle (C9). Moreover, miR-92b-3p regulates PTr2 proliferation and migration via the *PFKM* gene (Figure 7); thus, inhibiting miR-92b-3p expression adversely impacts mouse embryo implantation.

## 4. Materials and Methods

### 4.1. Sample Tissue Collection

In this study, healthy Large Yorkshire sows of similar age and genetic background were selected and treated to attain simultaneous estrus. They underwent artificial insemination after estrus was observed (recorded as day 0); one group was subjected to sham insemination (non-pregnant group), while the other group was subjected to frozen fresh semen insemination. Day 1 post-insemination was identified as the first day of pregnancy. Uteri were harvested on day 9 of estrus and days 9, 12, and 15 of gestation, respectively. The uterus was incised longitudinally on its inner side, and endometrial tissue was collected. It was immediately snap-frozen in liquid nitrogen and transferred to a −80 °C refrigerator for storage. All procedures involving animals were conducted under a protocol approved by the Ethics Committees of the Laboratory Animal Center of South China Agricultural University (approval No. SYXK-2019-0136).

### 4.2. FISH Assay

The FISH method was employed to detect the localization and relative quantification of miR-92b-3p in endometrial and embryonic tissues. The probe sequence of miR-92b-3p was 5′-UAUUGCACUCGUCCCGGCCUCC-3′Cy3 with red labeling. The basic procedure was as follows: the tissues were washed and immediately placed into 4% paraformaldehyde (Servicebio, Wuhan, China) for more than 12 h. After the fixation was completed, the sections were dehydrated by gradient alcohol embedded in wax, and then incubated for 2 h at 62 °C. The sections were dewaxed and dehydrated in turn. After fixation, the sections were dehydrated using gradient alcohol, embedded in wax, and subsequently incubated for 2 h at 62 °C. The sections were dewaxed and dehydrated in turn. After natural drying, the sections were boiled in the repair solution for 10–15 min, cooled naturally, digested with proteinase K (20 μg/mL) dropwise at 37 °C, rinsed with pure water, and washed thrice with PBS (Servicebio, Wuhan, China) for 5 min each time. Pre-hybridization solution was added dropwise to the sections, followed by incubation at 37 °C for 1 h. The pre-hybridization solution was removed, and the hybridization solution containing the probe was added dropwise for overnight hybridization in a 37 °C thermostat. After incubation, the hybridization solution was washed off, and the sections were washed with 2 × SSC (Servicebio, Wuhan, China) at 37 °C for 10 min, then with 1 × SSC at 37 °C for 2 × 5 min, and lastly with 0.5 × SSC at room temperature for 10 min. The sections were incubated for 8 min in a light-proof environment following the dropwise addition of DAPI (Servicebio, Wuhan, China) staining solution, rinsed, then sealed with the dropwise addition of anti-fluorescence quenching sealer (Servicebio, Wuhan, China). The sections were placed under a fluorescent microscope (Nikon, Tokyo, Japan) for visualization and image acquisition.

### 4.3. Cell Culture and Transfection

PTr2 cells were provided by Dr. Yulong Yin from the Institute of Subtropical Agriculture, Chinese Academy of Sciences. PTr2 cells were isolated form porcine filamentous embryos at day 12 of pregnancy and have been extensively characterized in previous published paper [43]. PTr2 were cultured in DMEM/F12 basal medium (Gibco, Grand Island, NY, USA) supplemented with 10% fetal bovine serum (Gibco, Grand Island, NY, USA), 0.5% insulin (YEASEN, Shanghai, China), and 1% penicillin-streptomycin (Gibco, Grand Island, NY, USA) at 37 °C with 5% CO2 in a constant-temperature cell incubator. When the cell density reached 80%, 0.25% Trypsin (Gibco, Grand Island, NY, USA) was utilized to digest cells and plates were seeded with PTr2 in preparation for subsequent experiments. PK-15 cells were cultured in DMEM/F12 basal medium (Gibco, Grand Island, NY, USA) supplemented with 5% fetal bovine serum (Gibco, Grand Island, NY, USA) and 1% penicillin–streptomycin (Gibco, Grand Island, NY, USA), and placed in a constant-temperature cell incubator maintained at 37 °C with 5% CO2. PTr2 cells in the logarithmic growth phase were inoculated into 6-well plates and cultured overnight until the cell density reached 50%–70%. miRNA mimics/antagomir/si-RNA/NC (GenePharma, Suzhou, China) were transfected with Lipofectamine 3000 Transfection Reagent (ThermoFisher, Waltham, MA, USA), following which they were cultured for 6–8 h in a cell incubator. The culture solution was changed after 6–8 h for subsequent experiments.

### 4.4. RNA Extraction and RT-qPCR

TRIzol (Ambion, Austin, TX, USA) was utilized to extract cell RNA, and RNA concentration was determined using a spectrophotometer. RNA was then reverse-transcribed into cDNA using a reverse transcription kit (TaKaRa, Kyoto, Japan). To establish the relative gene expression levels, a qPCR reaction system was implemented via a fluorescent quantitative PCR instrument. ssc-miR-92b-3p and gene expression levels were calculated according to the 2 −ΔΔCt formula using U6 and β-actin as internal controls.

### 4.5. Cell Counting Kit-8 (CCK-8) Assay

CCK-8 (YENSEN, Shanghai, China) was employed to detect the proliferation of cells in various periods. A 10 μL measure of CCK-8 solution was added to each well of a 96-well plate, and the plate was placed in a 37 °C cell incubator for 2 h. Each well’s optical density (OD) at 450 nm was measured using a multifunctional enzyme marker.

### 4.6. EdU Assay

The BeyoClickTMEdu-555 Cell Proliferation Assay Kit (Beyotime, Shanghai, China) was utilized to evaluate cell proliferation status at various time points post-transfection. The prepared 2× Edu working solution was added to the cell culture plate (96-well plate) at 48 h after transfection, followed by incubation at 37 °C for 2 h in a 5% CO_2_ cell culture incubator. Once Edu labeling was complete, the culture solution was removed, cells were washed twice with DPBS (Servicebio, Wuhan, China) for 3 min each time, 4% paraformaldehyde (Servicebio, Wuhan, China) was used for fixation at room temperature for 15 min, followed by washing with DPBS thrice, and cells were permeabilized for 10 min using a cell permeabilization solution. This was followed by washing twice with DPBS, the addition of 70 μL of prepared Click reaction solution to each well (96-well plate), and incubation for 35 min at room temperature and without light. The Click reaction solution was aspirated and discarded, and cells were washed twice with DPBS and incubated with DAPI (Servicebio, Wuhan, China) for 8 min at room temperature to stain cell nuclei. Subsequently, the DAPI solution was aspirated and discarded, and cells were washed twice with DPBS. Fluorescence detection was then performed.

### 4.7. Cell Wound-Healing Assay

After cells were transfected for 48 h, linear scratches were made vertically on 6-well plates with a large pipette tip, and the cell culture medium was changed to serum-free medium. Cell migration and scratch healing were visualized and photographed at 0 and 12 h. Three randomly selected visual fields were used to calculate the healing area using Image J software to obtain the cell migration rate.

### 4.8. Western Blotting

RIPA Lysis Buffer (CWBIO, Beijing, China) containing protease inhibitors was used to extract proteins. Samples were denatured by heating, proteins were separated via SDS–PAGE Gel (EpiZyme, Shanghai, China) electrophoresis and transferred onto PVDF membranes (Merck Millipore, Darmstadt, Germany), sealed with 5% skimmed milk (BD, Franklin Lakes, NJ, USA) for 2 h in a shaker at room temperature, washed with TBST, and incubated overnight at 4 °C with *PFKM* (Abcam, ab154804, 1:1000, Cambridge, UK) and Tubulin (Servicebio, GB11017, 1:1000, Wuhan, China) antibodies. Next, the samples were washed thrice with TBST and incubated with the secondary antibody (HRP-conjugated Goat Anti-Rabbit IgG, BBI Life Sciences D110058, 1:10,000, Shanghai, China) at 37 °C for 1 h. The target protein bands were visualized using chemiluminescent solution (CWBIO, Beijing, China), and the relative protein levels were quantified via Image J software.

### 4.9. Dual-Luciferase Reporter Gene Assay

Using PITA, miRanda, and RNAhybrid, the target gene of miR-92b-3p was predicted, the porcine *PFKM* gene’s 3′-UTR binding site that binds to miR-92b-3p was cloned into the pmirGLO vector (Promega, USA), and the mutant plasmid was constructed by targeted mutagenesis. PK-15 cells were inoculated into cell culture plates at 1 × 104 per well, and miR-92b-3p-mimics and dual-luciferase reporter plasmids (pmirGLO-*PFKM*-WT, pmirGLO-*PFKM*-MUT) were transfected into PK-15 cells using Lipofectamine 3000 transfection reagent when cell confluence reached 60–80%. The solution was changed after 6 h of transfection. After 24 h of transfection, the treated cells were harvested and assessed for luciferase activity using the Dual-Luciferase Assay Kit (YENSEN, Shanghai, China).

### 4.10. Intrauterine Injection in Mice

We selected 6–8-week-old ICR mice as an experimental model, and after mating, vaginal plugs were observed on the first day of gestation. At day 3 of gestation, pregnant mice were anesthetized, and 10 μL of 20 μM miR-92b-3p antagomir solution and 10 μL of DEPC water (GeneStar, Nanjing, China) were injected into both uterine horns; the mice were sutured and placed in a 37 °C environment for awakening. Mice were dissected on day 7 of gestation to observe the embryo attachment rate.

### 4.11. Statistical Analysis

All data were expressed as the mean ± standard deviation of at least three independent experiments, and the data were analyzed and plotted using the GraphPad Prism 8.0 software. T-test for independent samples was used for comparisons between two groups, with *p* < 0.05 indicating statistically significant differences and *p* < 0.01 indicating highly statistically significant differences.

## Data Availability

Not applicable.

## Supplementary Materials

The following supporting information can be downloaded at: https://www.mdpi.com/article/10.3390/ijms232416138/s1.
