# Supplementary figures and images for: Ssc-miR-92b-3p Regulates Porcine Trophoblast Cell Proliferation and Migration via the PFKM Gene
Source: Int J Mol Sci. 2022 Dec 17;23(24):16138. doi: 10.3390/ijms232416138 (PMC9784024; doi:10.3390/ijms232416138)

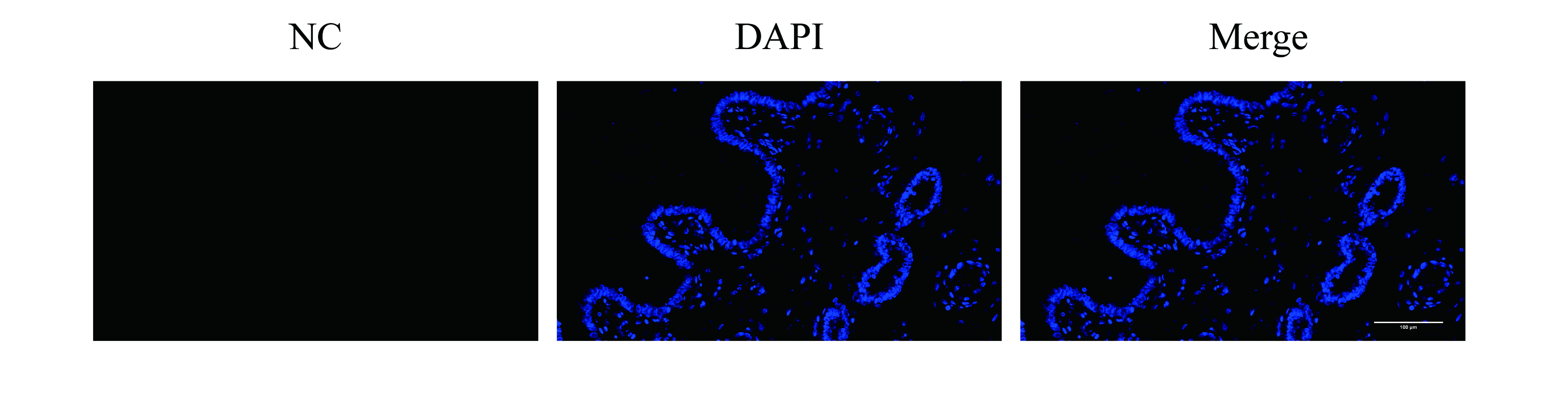

Supplement: Supplementary file 1 [file ijms-23-16138-s001.zip › Supplement Figure S1.tif]
